# *In silico* and *in vivo* analyses of the mutated human tissue plasminogen activator (mtPA) and the antithetical effects of P19 silencing suppressor on its expression in two *Nicotiana* species

**DOI:** 10.1038/s41598-018-32099-6

**Published:** 2018-09-19

**Authors:** Mahshid Amiri, Mokhtar Jalali-Javaran, Raheem Haddad, Parastoo Ehsani

**Affiliations:** 1 Department of Biotechnology and Plant Breeding, Faculty of Agriculture, Tarbiat Modares University (TMU), Tehran, Iran; 2 Agricultural Biotechnology Department, Imam Khomeini International University, Qazvin, Iran; 3 Department of Molecular Biology, Pasteur Institute of Iran (IPI), Tehran, Iran

## Abstract

Human tissue-type plasminogen activator is one of the most important therapeutic proteins involved in the breakdown of blood clots following the stroke. A mutation was found at position 1541 bp (G514E) and the mutated form was cloned into the binary vector pTRAc-ERH. *In silico* analysis showed that this mutation might have no significant effect on the active site of the tissue plasminogen activator enzyme. Accordingly, zymography assay confirmed the serine protease activity of the mutated form and its derivatives. The expression of the mutated form was verified with/without co-agroinjection of the *P19* gene silencing suppressor in both *Nicotiana tabacum* and *N*. *benthamiana*. The ELISA results showed that the concentration of the mutated form in the absence of P19 was 0.65% and 0.74% of total soluble protein versus 0.141% and 1.36% in the presence of P19 in *N*. *benthamiana* and *N*. *tabacum*, respectively. In *N*. *tabacum*, co-agroinjection of P19 had the synergistic effect and increased the mutated tissue plasminogen activator production two-fold higher. However, in *N*. *benthamiana*, the presence of P19 had the adverse effect of five-fold reduction in the concentration. Moreover, results showed that the activity of the mutated form and its derivatives was more than that of the purified commercial tissue plasminogen activator.

## Introduction

**Figure 1:**
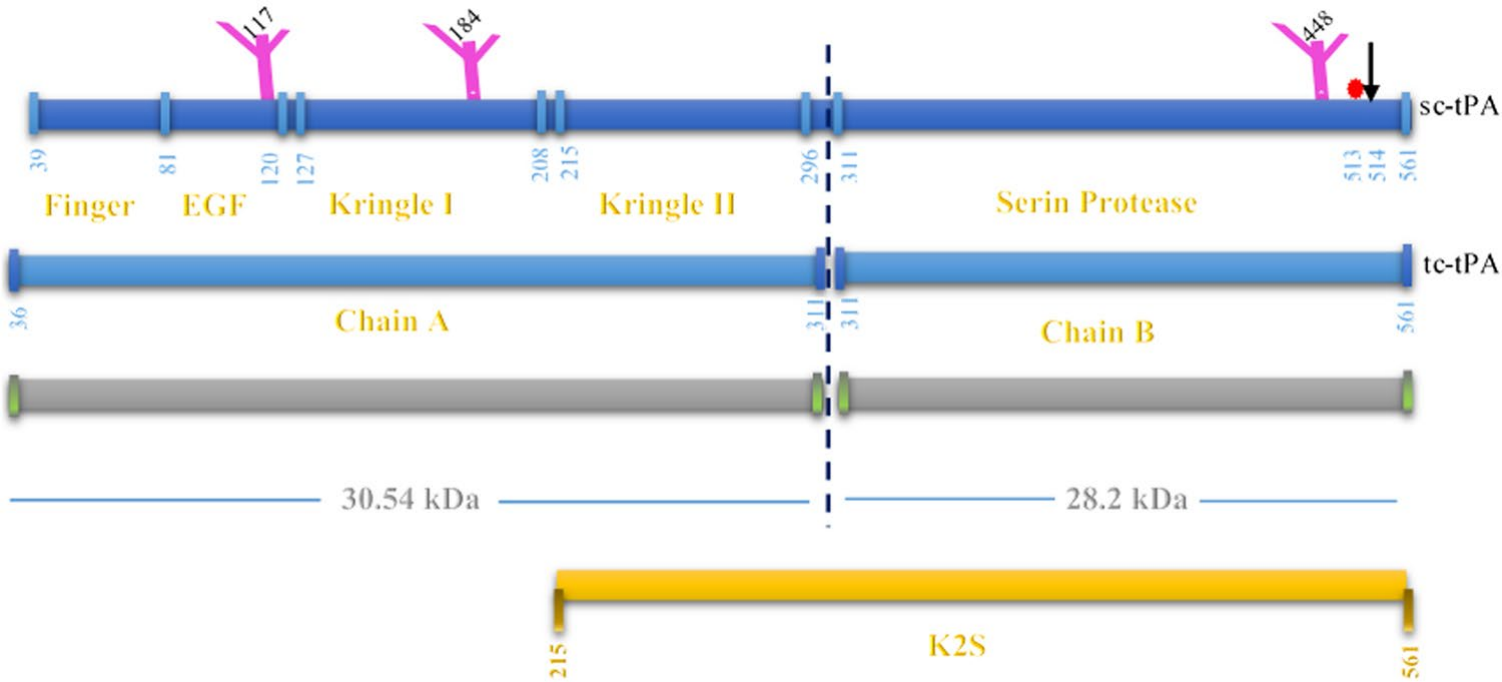
Schematic representation of the various molecular forms of t-PA.

Tissue plasminogen activator (tPA) is a serine protease with a molecular mass of ~64 kDa^1^. It is composed of five structural domains: a finger region, an epidermal growth factor-like subdomain, two kringle domains, and the catalytic domain^2^. This protein is responsible for the fibrinolysis activation through the conversion of the zymogen plasminogen to plasmin, resulting in the degradation of the fibrin network throughout clot dissolution^3^. The mentioned characteristic has taken into the account in the treatment of cardiovascular and cerebrovascular occlusion^4,5^. It has been recognized that the multiple variants of tPA have been identified even in the highly purified extraction. Natural tPA variants include the type I and II glycosylated forms and the single- and two-chain forms^5^. The different studies have reported tPA forms with the different molecular weight in the different tissues^6–9^. The active site of the protease is at position 513. Therefore, any novel mutation around this area could have a positive or negative impact on the activity of the protein which could be very important for the pharmaceutical industry (Fig. 1).

Considering the high importance and expensiveness of tPA, the recombinant tPA has been expressed in a wide range of the different expression systems like bacteria, fungi, insect cells, transgenic animals, and plants^5^. In bacteria, the production of the recombinant multi-disulfide proteins like tPA is usually complex due to the improper folding and accumulation in the misfolded structure^10–12^. The recombinant tPA expressed in the insect cells and *Saccharomyces cerevisiae* has shown the hyperglycosylation and the incorrect folding^13,14^. As a result of the high cost of the cell culture media and the virus and prion contamination, the mammalian cells are not an ideal system for the tPA expression^15–17^. The recombinant tPA has been expressed stably in both nucleus^15,16,18^ and chloroplast^17^ of plant *Nicotiana tobacum*. However, the level of the expression in the transgenic plants is not promising.

The plant stable and transient expression systems are two alternatives that have been developed for the production of pharmaceutics in the plants^19^. The transient expression system benefits from several advantages such as extremely rapid expression of the recombinant protein in less than a week, the lack of the generation and selection of transgenic plants^20–22^, the lack of the positional effects on the gene expression^21,23^, the simultaneous production of the multiple recombinant proteins, low environmental concerns, and the ease of scale-up^24^.

Among transient expression systems, the infiltration of the plant tissue with *Agrobacterium* (agroinfiltration) has developed rapidly^25^. Agroinfiltration also offers other advantages like flexibility, the ease of simultaneous expression of multiple genes in the same cell^26^, and its application in the industrial scale^21^. A major drawback of this technique is the post-transcriptional gene silencing (PTGS) as a limiting factor for the production which could be overcame by the P19 protein, a plant virus encoded silencing suppressor protein^26,27^. There are few reports on using P19 to enhance the recombinant protein expression level in *N*. *tabacum* such as the increased expression level of Hepatitis C virus (HCV) by over four-fold^28^ or the increased accumulation level of GFP^29^. On the contrary, there are some reports that P19 does not affect the expression^30^ or decreases the amount of the expression in *N*. *benthamiana*^31,32^. Therefore, implementing the P19 protein should be examined for each recombinant protein and cultivar independently.

The *in silico* data on the prediction of the expression and the activity of an enzyme is mostly based on the mammalian/prokaryotic expression environment. This is the first study in which the *in silico* prediction of a mutated enzyme activity is analyzed in the plant. Such *in silico* (computational) studies have the potential to reduce the guesswork drastically and to facilitate the development of the optimal biobetter since these studies inspect the validation of the complex structure with the best binding mode to design the biobetters^33^. A biobetter refers to a recombinant protein drug with structural and/or functional changes which improve them in one or more characteristics compared to the original^33–35^. To test the feasibility of producing a higher level of the new mutated form of the tPA protein, we transiently expressed the recombinant full-mtPA via *Agrobacterium*-mediated system in both *N*. *benthamiana* and *N*. *tabacum* plants for the first time. The *in vitro* activity of the mutated recombinant protein has been shown that the produced recombinant mtPA is active through *in vitro* analyses. Moreover, the impact of P19 silencing suppressor has brought about a completely different response on the production of mtPA in the different *Nicotiana* species.

## Materials and Methods

### Construct Preparation

The full-length human t-PA (GenBank accession number I01047) received from Dr. Mahboudi lab^36^ has been inserted in the binary expression vector pTRAc-ERH which has been kindly provided by Dr. R. Fischer (Fraunhofer Institute for Molecular Biology and Applied Ecology, Aachen, Germany).

**Figure 2:**
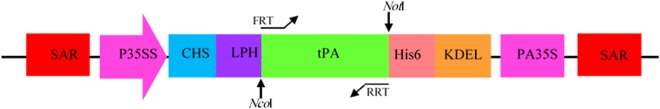
Schematic overview of the pMA2 construct (pTRAc-tPA-ERH).

The tPA has been amplified from TA-tPA vector (pMA1^37^) by primers containing *NcoI* and *NotI* sites (FtPA: 5′ ATATTCCATGGATGCAATGAAGAGAGG 3′; RtPA: 5′ ATAAGAATGCGGCCGC CGGTCGCATGTTGTCA 3′). The produced fragment has been digested by the *NcoI* and *NotI* restriction enzymes and cloned into the regarding sites in pTRAc-ERH, forming pTRAc-ERH-tPA (pMA2) (Fig. 2)^37^. Cloning of the gene has been confirmed by the colony PCR, double digestion, and sequencing.

### *In silico* Study

The assessment of the sequencing results has demonstrated a random point mutation in nucleotide 1541, a point mutation from guanosine to adenosine, in the active domain of tPA (Supplementary Fig. S1). To study protein structure, the consensus sequence has been translated into the protein by using Editseq software (Lasergene 7, DNASTAR Inc., Madison, WI, USA). MODELLER 9.14 (http://salilab.org/modeller/) software has been used to construct 10 homology models by employing the crystal structure of the tPA catalytic domain (PDB 1RTF) as template. To select the best model, the structure validation has been carried out by utilizing Structure Analysis and Verification Server (SAVES) (http://services.mbi.ucla.edu/SAVES/) via VERIFY3D^38^ and ERRAT^39^ programs. Then, GROMACS is applied as module to minimize energy resulting in the protein stability, by which the model is tested in water solvent to simulate the real and biological environment. The molecular docking of tPA and mtPA and plasminogen (PDB 4DCB) has been conducted using the HADDOCK web server) http://www.nmr.chem.uu.nl/haddock) to carry out comparative analysis between native and mutant forms. Dependent upon the quality of docking results based on the highest-ranking cluster, the native and mutant forms have been compared utilizing LigPlot v.1.4.5^40^ to ensure their poses are biologically acceptable.

### Preparing competent *Agrobacterium tumefaciens* for the electroporation

*A*. *tumefaciens* GV3101 strain with the pMP90RK helper plasmid GV3101::pMP90RK cultures grew to an OD600 of 0.8–1.0. All the following steps had to be done on ice at 4 °C. The medium chilled for 20 minutes and centrifuged at 4,000 g for 10 min. The pellet was resuspended in 100 ml of water and left for 15 minutes before the centrifugation. The obtained pellet was resuspended in 50 ml of water and centrifuged for 10 min twice. Then, the pellet was resuspended in 25 ml of the cold sterilized solution (272 mM sucrose and 15% glycerol) and left for 5 minutes and centrifuged. The final pellet was suspended in 2 ml of sucrose-glycerol and stored at −70 °C in 100 µl aliquots. In our study, all the chemicals and antibiotics purchased from Sigma (US) and Merck (Germany) companies.

### *Agrobacterium* Transformation

100 μl of GV3101::pMP90RK was electroporated with 100–200 ng of pMA2. Electroporation was carried out utilizing Eppendorf 2510 set at 2 kV, 25 μF, and 200 ohm. Electroporated cells were cultivated in 1 ml TSB medium (Conda, Spain) for 3–4 h in a shaker at 28 °C, 180 rpm before plating on TSB agar medium containing 100 μgml^−1^ Carbenicillin, 100 μgml^−1^ Rifampicin, 25 μgml^−1^ Kanamycin, and 25 μgml^−1^ Gentamycin for 72 h at 28 °C. The transformed *Agrobacterium* colonies were confirmed by gene-specific PCR.

### Agroinjection

The transformed *A*. *tumefaciens* was incubated overnight in TSB medium with the mentioned antibiotics at 28 °C. The overnight culture was deposited by the centrifugation at 5,000 g for 15 min, suspended in an agroinjection buffer^41^, and diluted to OD600 = 0.3. To suppress the post transcriptional gene silencing, the equal ratio of the agrobacteria containing pMA2 and agrobacteria containing pCAMBIA 1304- *P19* (a gift from Dr. Ehsani, Department of Virology, Pasteur Institute of Iran, Tehran, Iran) was prepared. The mixture was injected into *N*. *benthamiana* and *N*. *tabacum* leaves using needle-free syringe in the back of the leaves and the plants were grown for four days at 16 h long daylight at 23 °C.

### Expression Analysis of mtPA at the RNA Level

Total RNA of 10 mg was extracted from the young tobacco infiltrated leaves using an RNA purification kit (Topazol plus, Iran) and treated with DNAse (Fermentas). The cDNA synthesis was carried out by a First Strand cDNA Synthesis Kit (Fermentas). RT-PCR was performed on cDNA by following mtPA specific primers:

FRT: 5ʹ-GGAAACAGTGACTGCTACTTTGGGAATGG-3ʹ

RRT: 5ʹ-TTGATGCGAAACTGAGGCTG-3ʹ

### Analysis of the Recombinant mtPA Protein Expression

The injected leaves (200 mg) were ground to a powder by the liquid nitrogen, suspended in 1 ml of a protein extraction buffer [50 mM Tris–HCl (pH 7), 2 mM EDTA, 0.1% (w/v) sucrose, 0.04% (v/v) 2-mercapthoethanol, and 2 mM PMSF], and homogenized. Supernatant was transferred to a fresh 1.5 ml microfuge tube after the centrifugation at 24,000 × g, 4 °C for 20 min and its total soluble protein concentration was measured by the Bradford technique^42^. Dot Blot Enzyme Immunoassay was conducted to confirm the presence of mtPA. The positive control (Activase^®^ (Alteplase), Genentech), 15 μg of total soluble protein of each sample, and the control plants were gradually spotted onto nitrocellulose, dried and blocked in 1% BSA in PBST for 1 h. Then, three washes were done using TBS-T for 10 min. The rabbit polyclonal antibody against tPA (ab28219, USA) was added 1:2000 for 1 h. After three washes, the goat anti-rabbit IgG-HRP antibody (Sc-2030, USA) was added to blot as 1:2000 dilution for 1 h and washed as above with PBST for 10 min. Immunoreactive proteins visualized by DAB (diaminobenzidine) and H_2_0_2_ solution.

The western blot assay was performed using 30 μg of total soluble protein loaded on 12% sodium dodecyl sulfate polyacrylamide gel (SDS– PAGE) and run on 20 Volts. Then, the protein bands were transferred onto the polyvinylidene difluoride (PVDF) membrane (Thermo Scientific, USA) utilizing the transfer buffer (48 mM Tris, 39 mM Glycine, 1.3 mM SDS, and 20% methanol; pH 9.2) in Trans-Blot SD semi-dry electrophoretic transfer cell (Bio-Rad, USA) apparatus. The PVDF membrane was blocked by 5% (w/v) nonfat skim milk powder in PBS at 4 °C overnight. The bands were developed as mentioned above.

### Quantitative Evaluation of the Transient mtPA Expression

45 ng of total soluble protein diluted in a carbonate/bicarbonate coating buffer (0.1 M, pH 9.6) was loaded into a 96-well polystyrene microplate in three experimental and two biological replicates and incubated overnight at 4 °C. The plate was washed three times using a phosphate-buffered saline with 0.05% Tween 20 (PBST) and treated by adding a blocking buffer (1% BSA in PBST) for 1 h at 37 °C. After washing with PBST, the dilution of the Rabbit polyclonal antibody against tPA (Abcam, USA) with a blocking buffer (1:2000) was added for 1 h at 37 °C. The plates were washed and loaded with Goat anti-Rabbit IgG HRP conjugate (Sc-2030) in PBST (1:2000) and incubated for 1 h at 37 °C. Following washing step, the solution containing 1% TMB (3,30,5,50-tetra-methylbenzidine), 0.01% H_2_O_2_, in 200 mM citrate buffer, pH 5 was added and incubated for 15–30 min at 37 °C. 1 N H_2_SO_4_ was added as stopping solution and read at 450 nm using a micro plate reader (BioTek, USA). The concentration of the protein has been calculated against the standard curve using the serially diluted commercial 60 kDa tPA (Activase^®^ (Alteplase), Genentech).

### Activity measurement of the transient mtPA expression

Zymography was performed to assess the plasminogenolytic activity of the transiently expressed mtPA. It was carried out as described before with the slight modification^43^. The 12% SDS poly acrylamide resolving gel has been supplemented with 0.1% (w/v) gelatin (Sigma, USA) and plasminogen (chromogenix, Italy) (10 μg/ml). Electrophoresis was run at a continual current of 10 mA at 4 °C. The residual SDS was washed sequentially twice by soaking the gel in the 2.5% Triton X-100 for 30 min on the shaker at RT, followed by rinsing the gel with distilled water. The gel incubation in 0.1 M Glycine/NaOH (pH 9.3) was carried out for 3 h at 37 °C. The gel was subsequently stained and destained via the Coomassie Brilliant Blue R-250 procedure.

### Quantification analysis of the transient mtPA expression

To investigate if this random point mutation could lead to producing a biobetter, we quantitatively compared the commercial tPA and plant-produced mtPA. Quantification of zymogram and western blot bands was performed using ImageJ software (National Institute of Health, Bethesda, MD, USA) and quantitative data was normalized by Microsoft Excel. Values were presented as mean ± SE from three independent replicates. The quantitative data were compared between the bands of mtPA of the lane T3-P19 and the commercial tPA using Microsoft Excel’s paired two-sample t-test for means.

## Result

### *In silico* studies

The *tPA* gene has been sequenced and the BLAST result with AAH95403.1 (identity score 99% and query coverage 100%) has illustrated a point mutation at the position 1541 in which a G to A transition has led to substituting Glutamate for Glycine at codon 514 (Supplementary Fig. S2), converting a nonpolar and hydrophobic amino acid into a polar and hydrophilic one which will be called mutated tPA (mtPA). As this missense mutation in codon 514 is situated exactly after the active site at codon 513 which is in the catalytic domain, it seems crucial to investigate the precise effect of the mutation on the structure and function of the recombinant protein.

For structural determination, the modelled structures have been prepared and shown that the amino acid position 196 is located one amino acid away from the catalytic site. These structures have been validated via objective function, ERRAT, and Verify3D. The analyses have inferred that model 1 which is allocated the parameter values with the minimum objective function^44^, the quality factor above 70% of ERRAT, and the model quality score of 97.81% with Verify3D with an average scoring above 0.3^45,46^ is the best one among the suggested models (Table 1, Supplementary Figs S3, S4).

**Table 1:** Evaluation and validation scores of models.

Models	Objective Function	ERRAT Quality Factor	Verify3D Score
Template		94.248	96.05%
Model 1	14028.0693	81.481	97.81%
Model 2	29082.4492	79.630	98.68%
Model 3	13392.3828	80.000	96.93%
Model 4	21148.4551	77.778	97.37%
Model 5	28772.8730	70.642	96.93%
Model 6	29074.4883	73.148	97.81%
Model 7	28919.8574	78.279	98.25%
Model 8	21264.9180	86.574	96.93%
Model 9	14370.3857	68.349	98.25%
Model 10	28412.4258	73.023	98.25%

**Figure 3:**
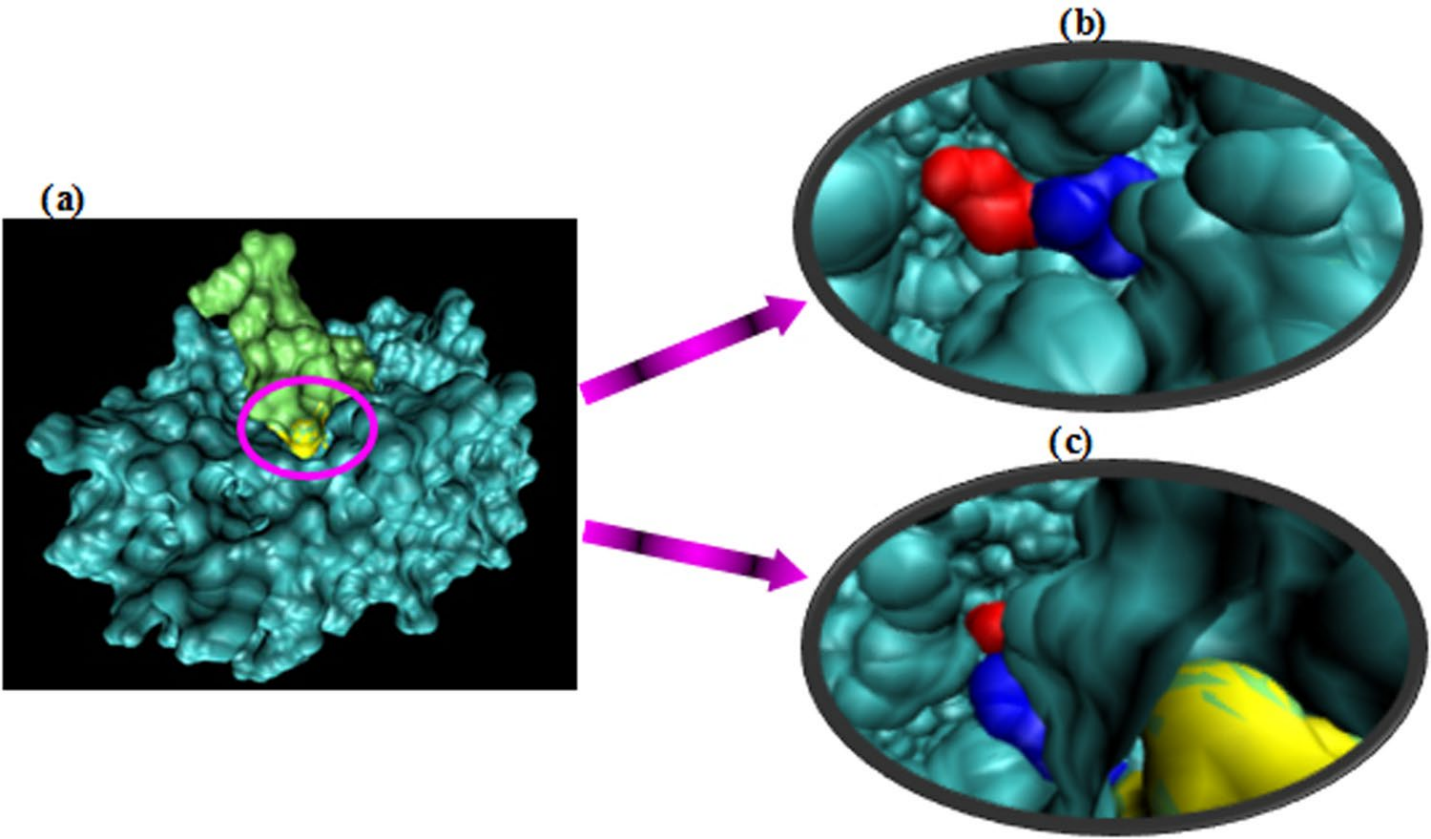
Surface representation of the interaction between the native form of tPA and plasminogen.

**Figure 4:**
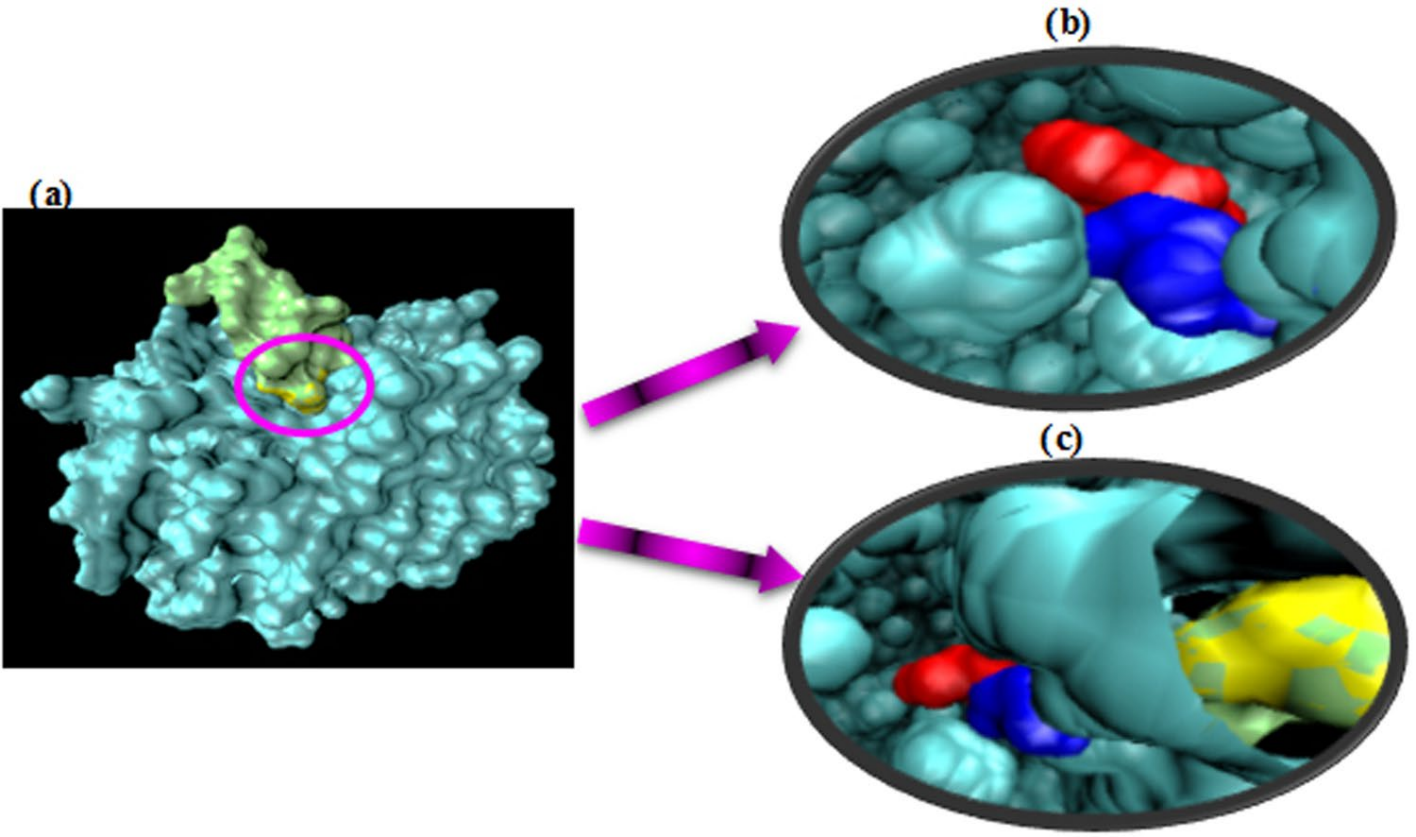
Surface representation of the interaction between the mutated form of tPA and plasminogen.

Prior to the protein-protein docking, the native and mutant complex structures have been subjected to the energy minimization. In order to visualize the role of the substituent at position 514, we have performed *in silico* molecular docking analyses between both native and mutant forms of tPA and plasminogen. The substitution of Glutamate to Glycine has not shown to affect the interaction between tPA and plasminogen in the mutated form. As it can be observed in the molecular docking results, the spatial interaction pattern between the native form of tPA and plasminogen (Fig. 3) is similar to the one between the mutated form of tPA and plasminogen (Fig. 4). The results show that the mutant type of tPA can interact with plasminogen as substrate.

**Figure 5:**
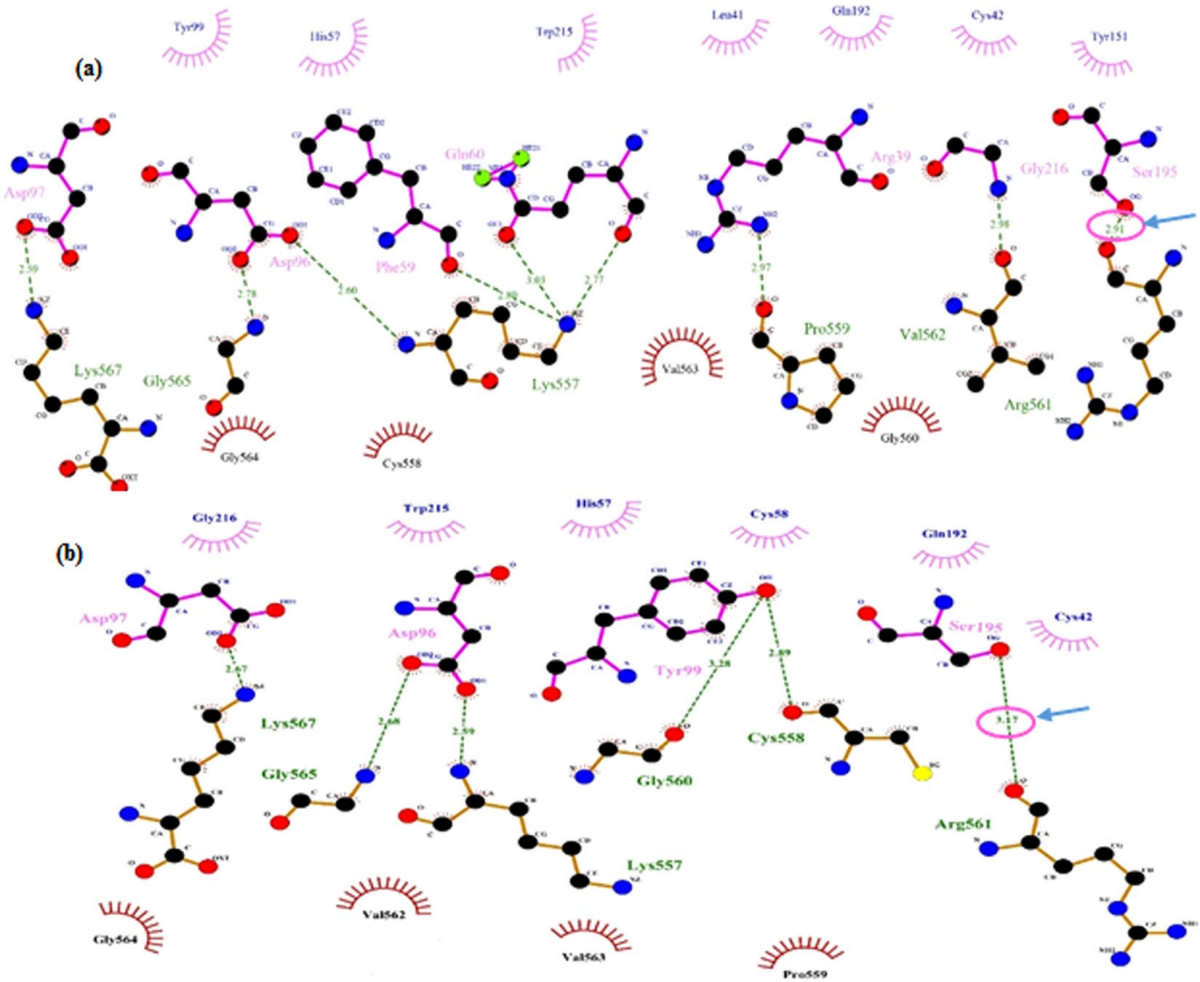
Ligplot diagram of the interactions at the protein–protein interface in (**a**) native form, (**b**) mutant form of tPA and plasminogen.

For more detailed assay, the protein-protein interactions have been schematically presented by LigPlot. As it is depicted in Fig. 5, several hydrophobic and hydrogen bonds have been formed. The amino acid residue Ser195 (C195 in this study) of the native form which plays a critical role in the recognition of the residues Arg561-Val562 of plasminogen has been found to have the similar pose in the mutant form. The mutant type of tPA has contact with the substrate surface, interacting with residues Arg561-Val562. In the native form, the relative distance of the interaction between Arg561 and Ser195 (C195) is 2.91 A°. The substitution of Glutamate has reduced its relative distance to 3.17 Å which it could cause the minor conformational change and affect the interaction. However, it should be considered that the overall conformation is similar and the 0.26 Å difference of the relative distance may not impose a spatial restriction to the plasminogen binding.

### Expression analysis of mtPA at the RNA level

**Figure 6:**
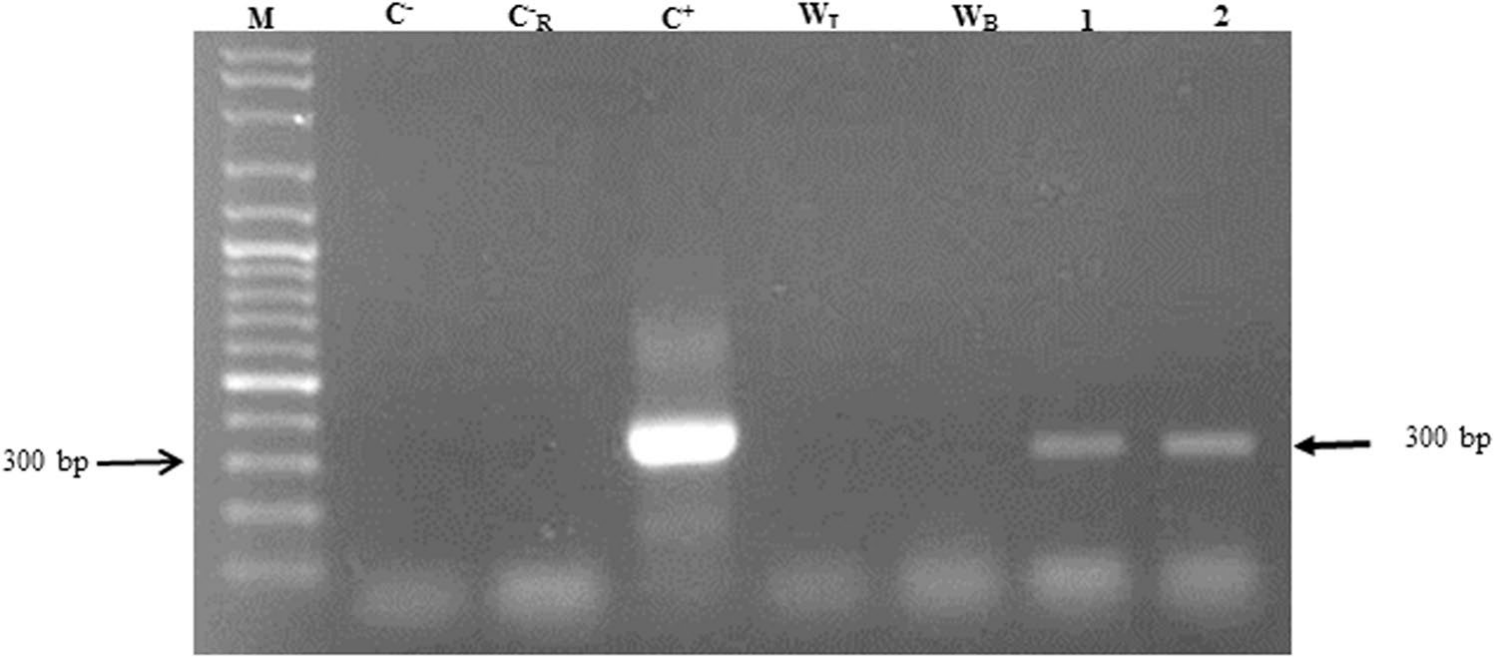
RT-PCR amplification of tissue plasminogen activator on RNA extracted from agroinjected leaves.

To confirm the expression of mtPA at the RNA level in the agroinjected leaves, mtPA treatment of each species was analyzed. RT-PCR resulted in a band of 300 bp corresponding to the size of the expected amplified transcript of mtPA in the agroinjected leaves (lanes 1 and 2), supporting the idea of mtPA expression. No RT reaction product was confirmed in the absence of the gene encoding mtPA in the injected leaves with *Agrobacterium* transformed with an empty pTRAc-ERH as a negative control (Lane W_T_ and W_B_) (Fig. 6).

### Detection of the transiently expressed mtPA

**Figure 7:**
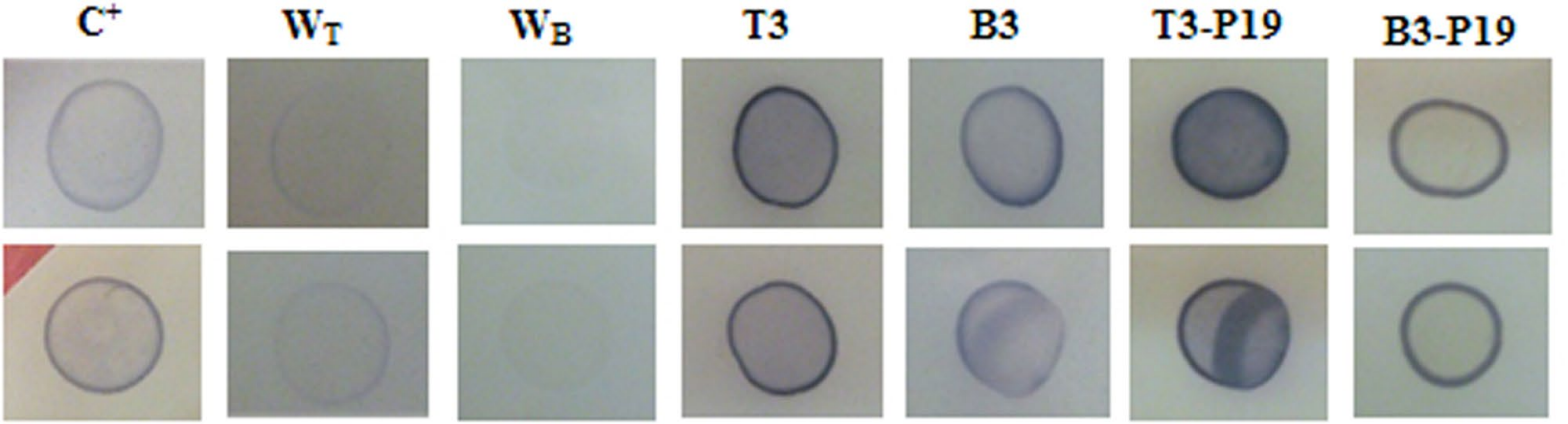
Dot blot analysis using tPA-specific antibody in two replicates.

To monitor the expression of the transient mtPA, the dot blot assay was carried out for all treatments of the negative control, mtPA + P19, and mtPA in both *N*. *benthamiana* and *N*. *tobaccum* on the same nitrocellulose paper. The assay showed the presence of the recombinant protein in the extract of the injected leaves in comparison to the negative control with the same volume of loaded TSP (1500 ng). As observed in Fig. 7, the color intensity was varied over all treatments, indicating the expression level of the recombinant mtPA. The produced level of mtPA in T3-P19 leaves was much more than that in B3-P19 and the slight difference was shown between T3 and B3 leaves.

**Figure 8:**
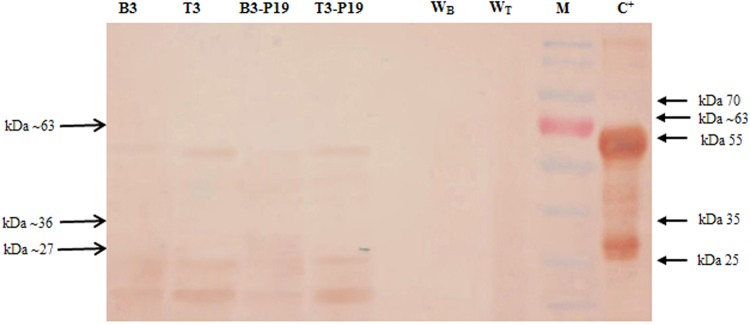
Western blot analysis with tPA-specific antibody.

To prove the presence of the transiently expressed mtPA, western blot with mtPA-specific polyclonal antibody has been conducted. As it can be observed, there are two distinct bands in the positive control Alteplase (63 & 36 kDa). The 63–68 kDa corresponds to the expected molecular weight of the full tPA protein and is also present in all plant treatments. The protein bands of approximately 27 kDa have also been observed in both *N*. *benthamiana* and *N*. *tabacum* extracts (T3-P19, T3, B3-P19, and B3). However, in the *N*.*tabacum* extracts, the amount of the full and processed mtPA proteins is more than that in *N*. *bentamiana* leave extracts. Interestingly, it has detected two bands about 36 kDa, which all belong to *N*. *tabacum* not *N*. *benthamiana*. In *N*. *bentamiana* leaves containing pMA2 without pCAMBIA-P19 (B3), the amount of mtPA proteins is more than that in *N*. *bentamiana* leaves with B3-P19 which is not well detectable. However, in *N*. *tabacum*, the amount of mtPA proteins is slightly more in treatments with pCAMBIA-P19 (T3-P19). The extraction from leaves injected with the *Agrobacterium* containing the untransformed pTRAc-ERH in *N*. *benthamiana* and *N*. *tabacum* has been used as the negative controls which do not show any interaction with the antibodies used for immunoblotting (Fig. 8). Normally, the protein divides into two different A and B chains in the same amount in our case (SDS-PAGE data has not shown), however, western blot shows two bands with the different amounts. The difference can be due to the antibody preparation used in our study or the fast processing of the mutated tPA because of the mutation effect on the protein conformation.

### Quantitative evaluation of the transient expressed mtPA

**Figure 9:**
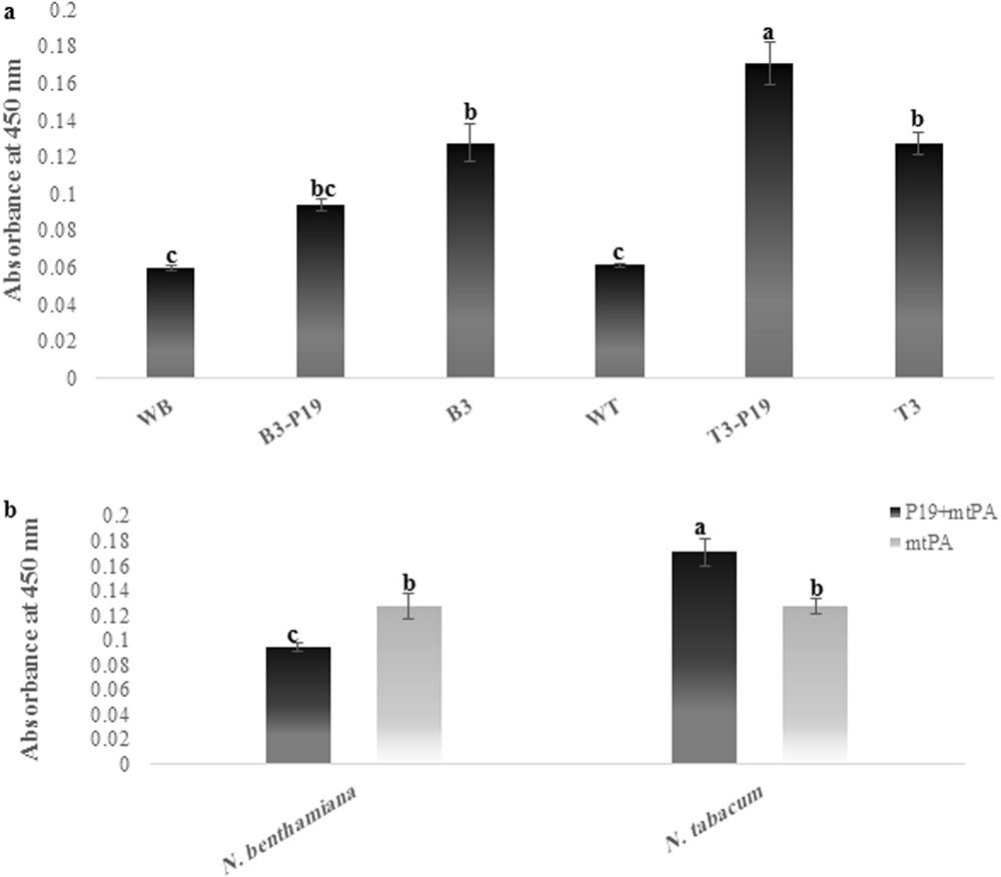
Transient expression of recombinant protein mtPA in agroinjected leaves of *Nicotiana* species.

The recombinant mtPA protein accumulation in the agroinjected leaf extracts of six treatments and the negative control plant has been analyzed by ELISA (Fig. 9a). To assess the significance of P19 as the gene silencing suppressor and plant species, three experimental and two biological replicates have been tested and the data has been subjected to the statistical analysis.

The results have shown that the gene silencing suppressor and plant species have had a significant impact on the mtPA level, moreover, the interaction between these two factors has been shown to be significant. The striking difference has been revealed in the expression level of mtPA between two different species *N*. *tabacum* and *N*. *benthamiana* agroinjected with and without pCAMBIA-P19 (Fig. 9b). *N*. *benthamiana* plants co-agroinjected with pCAMBIA-P19 and pMA2 have revealed the decreased production of mtPA compared to the plants agroinjected with pMA2 alone. Maximum level of mtPA has been reached 0.65% of TSP in *N*. *benthamiana* plants in the absence of the P19 co-expression. Inversely, the mtPA accumulation level has decreased to 0.14% of TSP in the presence of post-transcriptional gene silencing effect of pCAMBIA-P19 vector. By contrast, the co-expression of P19 results in the increased accumulation level of mtPA in the *N*. *tabacum* plants. In *N*. *tabacum*, the production level of mtPA with and without pCAMBIA-P19 vector co-agroinjection have been 1.36% and 0.74% of TSP, respectively. The leaves injected with the native *Agrobacterium tumefaciens* (with untransformed plasmid) are counted as a negative control.

### Activity measurement of the transient expressed mtPA

**Figure 10:**
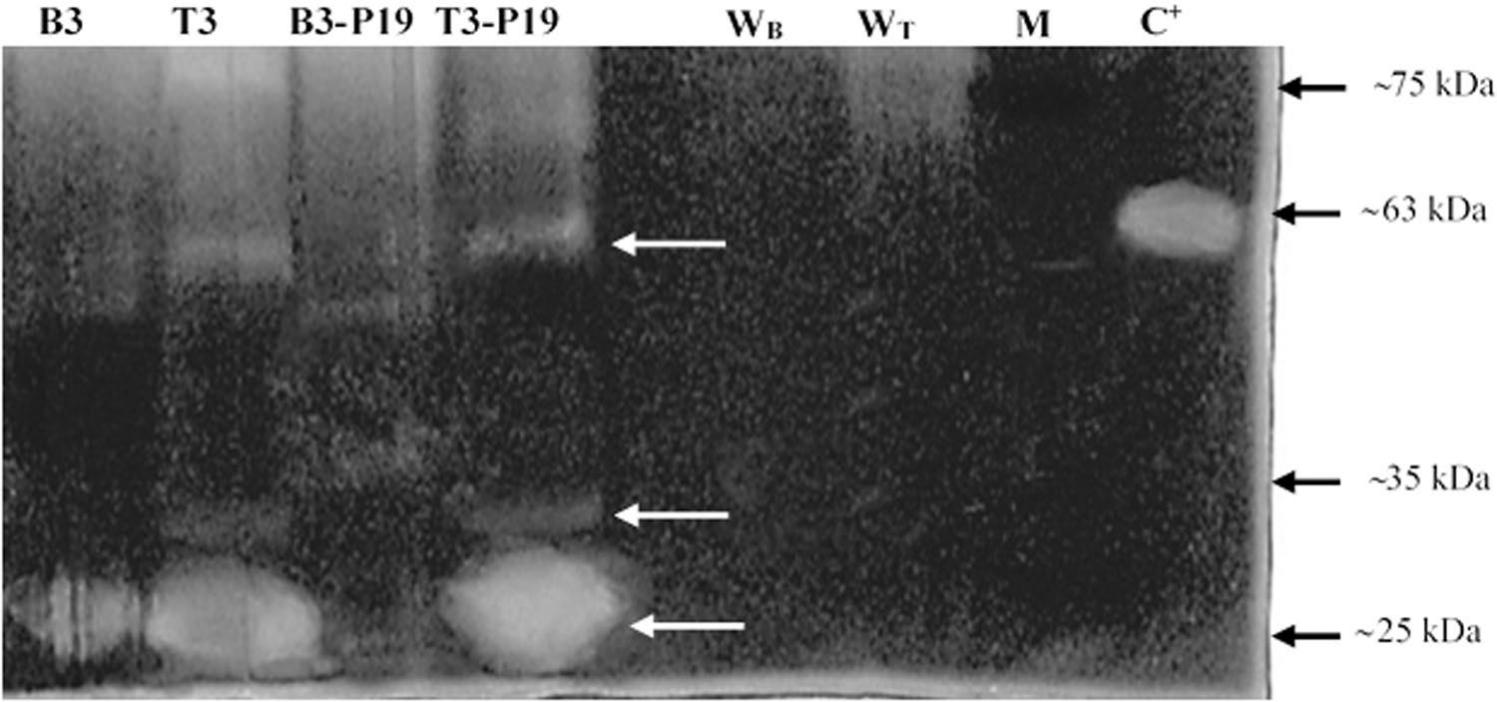
Zymography assay on extracted protein of injectd leaves.

To assess the proteolytic activity of the transiently expressed mtPA, the gelatin zymography assay was performed for two times. As observed, there is one clear band with a mass of about 63 kDa in the positive control while there is no evidence of the plasminogenolytic activity in the negative control of both *N*. *benthamiana* and *N*. *tobacum*. The proteolytic bands with the molecular masses of approximately 63 kDa are compatible with the full length tPA produced in both *N*. *benthamiana* and *N*. *tobacum*. Intriguingly, there are other lower molecular weight forms in mtPA + P19 and mtPA treatments of *N*. *benthamiana* (B3-P19 and B3), which can be the full length form of mtPA without one glycosylated site. The results have shown that there are the lytic activities in approximately 27 kDa fragments more than others. The mentioned 36 kDa fragments of the western blot have not been observed in this assay, which can indicate these fragments do not have the lytic activity, therefore, they can be the chain A fragments with two glycosylated sites. In addition, a decrease in the proteolytic activity of the plant extract is shown in *N*. *benthamiana* injected with pMA2 and pCAMBIA-P19 *Agrobacterium* versus non pCAMBIA-P19 treated leaves (Fig. 10).

### Quantification analysis of the transient mtPA expression

**Figure 11:**
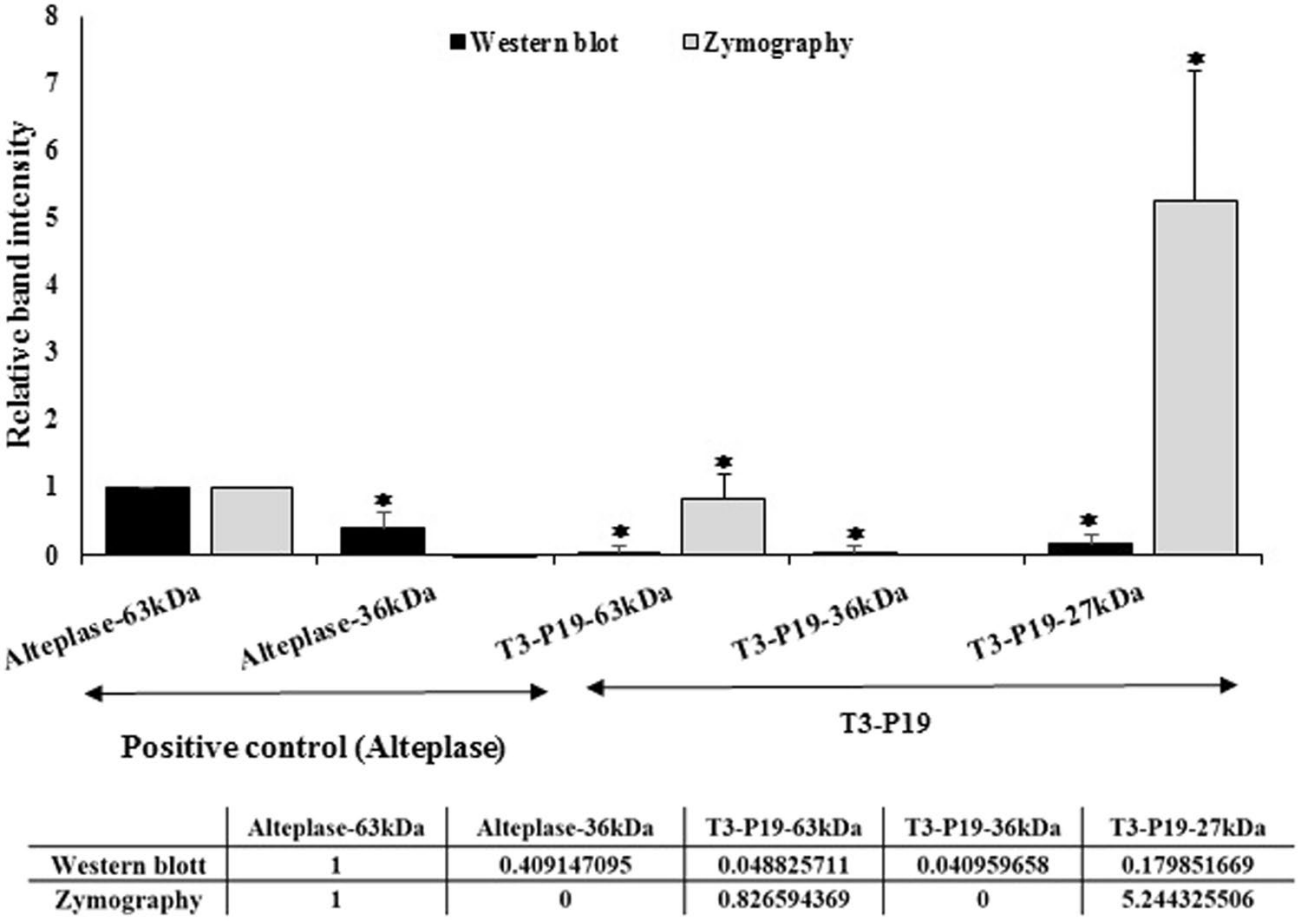
Quantitive data from zymogram and western blot comparing the commercial tPA vs. the mutated tPA.

In order to investigate the capacity of mutated tPA as a biobetter, the zymogenic activity of mtPA was quantified. The activity profile showed the significant difference between commercial tPA and mtPA (Fig. 11). We observed a 5.0-fold increase in the activity of the 27 kDa of the T3-P19, as opposed to the decrease in the activity of the 63 kDa of T3-P19 in comparison with the commercial tPA (Alteplase). The protein profile obtained from western blot revealed a different protein accumulation for each fragment. However, we did not observe the correlation between the protein accumulation and activity (Fig. 11).

## Dicussion

The tissue plasminogen activator is one of the most important pharmaceutical proteins in the treatment of the blood clots in human. Therefore, it has been expressed in the different prokaryotic and eukaryotic systems including plants. It is worth mentioning that a variety of tPA proteins have been found in the different human tissues.

Here, a mutated form of the full length *tPA* gene has been *in silico* studied. Then, it has been cloned into the plant cells and its biological activity has been investigated. In plant research, there is not much work performed on the experimental validation of *in silico* studies such as the interaction between the recombinant mutated protein and its substrate. This work could help to confirm the application of *in silico* studies in the plant biotechnology.

The mutation has been identified by sequencing to be at Glycine 196 (C196) which is located one residue after Serine195 (C195), the crucial residue which is responsible for serine protease activity of 5th domain of tPA^47^.

The present study has striven to assess whether this spontaneous missense mutation of Glycine (hydrophobic amino acid) to Glutamate (charged amino acid) has an impact on the catalytic activity of the neighboring residue. Therefore, the protein–protein docking has been done to solve the question by modeling the direct physical interactions of the residues^48^ and shown that the interaction between mtPA and plasminogen is still maintained. Moreover, the *in silico* studies have been further verified by running the *in vitro* experiments on the activity of mtPA.

Primarily, the result of the RT-PCR has shown that the mRNA of the mutated form is produced in both species. Dot blot analysis has shown the presence of the correct antigenic conformation of mtPA in both *Nicotiana* species using the polyclonal antibody against tPA.

The expression of tPA in mammalians has shown a single chain of 64 kDa in pig heart^7,49^ or 72 kDa in the human melanoma cells^6,9^. Moreover, hairy roots of oriental melon^50^ and *N*. *tabacum*^15,16,18^ plants have also produced tPA in the different molecular weight ranges.

In general, in eukaryotic cells, the full length tPA (63 kDa) could be further split by plasmin^6,9^, trypsin and other serine proteases^9,51^ into the equal amounts of two chains of A 30.54 kDa and B 28.2 kDa. These fragments can be glycosylated to (A 33.54, A 36.54 kDa, and B 31.2 kDa) proteins which are cross-linked by a disulfide bond. The traversing of proteins in the ER can result in the hyper mannose glycosylation that increases their size^52–54^. In our study, there is a possibility that the A fragment is hyper glycosylated that produces a 36 kDa fragment while the B fragment is not glycosylated.

It is likely inferred that the observed multiple bands in western blot are the result of the natural processing of tPA in eukaryotic cells and different glycosylation patterns^5^. Our observation is in accordance with 31 kDa to 68 kDa bands of tPA observed in the hairy roots of the oriental melon^50^. However, the presence of KDEL at the C-terminal of mtPA protein and the cleavage site of the full length protein have resulted in the retaining of both full length protein and B fragment in the ER. Meanwhile, the A fragment has been further transferred to other compartments of the secretory pathways and degraded^55^. The unequal amounts of the A and B protein bands can be clearly observed in western blot and the presence of high amount of the B fragment has been confirmed using the zymogram.

The processing rate of mtPA is much higher than that of tPA proteins because western blot and zymogram have shown that most of the fragments in mtPA are in 27 and 33 kDa forms. However, the same immunochemical experiments on the expressed non mutated tPA protein in plants^15,16^ and animals^6^ have revealed 63 kDa form which has been found to be more abundant than the two other 25 and 36 kDa derivatives.

The activity recognition of the protein bands is mostly done by the hydrolysis of plasminogen in a zymogram gel^8,56^. Zymography assay has revealed that the 63 kDa band of the mutated form is functioning like the commercial protein (Alteplase) (Fig. 10). Soleimani *et al*.^36^ cloned and expressed the same tPA coding sequence (GenBank accession number I01047) in *Leishmania tarentolae* without (G514E) mutation. They showed the 25, 30, and 65 kDa protein bands were present and the most active protein was the 65 kDa. The 25 and 30 kDa could be the glycosylated and non-glycosylated forms of the serine protease fragments which were equally active in the zymogram experiment, however, the amount was less than 65 kDa^36^. Non mutated tPA protein was also expressed in the *N*. *tabacum* plant^15,16,37^. The zymography experiment of the plant produced mtPA has confirmed the results of Soleimani *et al*. (2010) by showing the same pattern for the mtPA protein. However, in our study, the approximately 27 kDa is very active in comparison to the tPA derivatives in above studies^15,16,36^. It is possible that the mutation near the active site serine 513 can affect the activity of the spontaneously mutated form of the 27 kDa fragment of tPA. These results are not limited to our findings; Pop *et al*. (2003) have substituted valine266, a non-polar and hydrophobic amino acid, to Glutamate, an acidic and polar amino acid in the center of the procaspase-3 dimer interface in zymogen, resulting in the increased enzyme activity up to ∼60-fold^57^. The missense mutation in our study (G514E) is similar to that of Pop *et al*. (2003) study (V266E). In addition, Pop *et al*. (2003) have shown that the replacement of V266 with histidine has terminated the activity of the enzyme. They have demonstrated that V266H causes the long distances > 20 Å and the dimer interface integrity is crucial for providing the proper active site conformation^57^. Therefore, the slight change in the interaction distance between the native and mutant forms of tPA with the substrate (0.26 A°) could lead to the optimized enzyme activity.

In addition, while in western blot, using the polyclonal antibody, the amount of the alteplase is more than the plant produced B fragment (27 kDa), The zymogram confirms that B fragment is several fold more active than the commercial alteplase. However, the comparison between the activity of the full length mtPA and Alteplase should be further investigated. These preliminary results on the enhanced activity of mtPA could increase the knowledge of tPA protein structure function for better drug design applications.

While the amount of the alteplase is more than that of the plant produced B fragment (27 kDa) in western blot, the zymogram qualitatively confirms that the B fragment is several fold more active than the commercial alteplase.

The quantitative comparison between the commercial tPA (Altaplase) and B fragment of mtPA revealed a 5.0-fold increase in the activity of B fragment in comparison with Altaplase, despite the fact that we have loaded 500 μg/ml pure tPA and 1640 μg/ml total soluble protein of the sample. However, we observed the protein accumulation in the 36 kDa fragments of the commercial tPA and mutated tPA without any activity, which showed they did not have any activity. Moreover, in spite of the low accumulation of fragments of the T3-P19 in the quantified data derived from western blot, the activity was significant. As mentioned above, the slight change in the interaction distance as a consequence of the point mutation might improve the functional characteristic of this recombinant protein leading to emerging a new candidate drug with more activity or a biobetter. These preliminary results of the enhanced activity of mtPA could increase the knowledge of tPA protein structure-function for better drug design applications.

There is an obvious disparity among *N*. *benatmiana* and other species as a result of genotype differences^58^, That is why the transient expression of the pharmaceutical proteins is preferably performed in leaves of *N*. *benthamiana*^27,31,59–61^.

In our study, the enhancing effect of P19 silencing suppressor on the mtPA expression has been also investigated in two *Nicotiana* species. The results have shown that mtPA has been expressed in both plants. However, P19 silencing suppressor is more effective in *N*. *tabacum* cv. Xanthi (Fig. 9b) compared to *N*. *bentamiana*. The ELISA assay has confirmed that the accumulation level of mtPA without and with P19 is 0.74% and 1.36% in *N*. *tabacum* versus 0.65% and 0.144% of TSP in *N*. *benthamiana*, respectively. Ha *et al*. (2017) interestingly showed that the expressed level of the human acidic fibroblast growth factor (aFGF) decreased approximately two-fold lower in the presence of P19 silencing inhibitor^32^. This result is in accordance with our antithetical results that the decrease of the expression level has been five-fold lower in *N*. *benthamiana*.

Moreover, co-agroinjection of P19 and mtPA in *N*. *tabacum* showed the two-fold increase in the expression level of the mtPA in comparison to the agroinjection of the mtPA treatment alone. This is in accordance with Mohammadzadeh *et al*. (2014) that co-expressed Hepatitis C core antigen with P19 in the same species and observed the increased expression of the recombinant protein from 0.004% to 0.022% of TSP^28^. Furthermore, our previous study achieved the two-fold increase of non mutated tPA using the P19 co-agroinjection^37^.

In western blot, the approximate 63 kDa band is present in all treatments, however, the recombinant mtPA bands are fading away in *N*. *bentamiana*, which it is clearly detectable in P19 co-agroinjected *N*. *bentamiana* leaves. Moreover, the protective ER environment has not supported the retained protein stability. Therefore, the 27 kDa protein has been completely vanished in the P19 co-expressed plant leaves in *N*. *bentamiana* and this is in contrary to the effect of P19 in *N*. *tabaccum*. The adverse effect of P19 accompanied with a protease (mtPA) in two species of *Nicotiana* seems to be a novel and interesting field of the research.

There is a possible toxicity of mtPA for *N*. *bentamiana*, resulting in the decreased accumulation of the protein in this species in comparison to *N*. *tabacum*. This is previously observed in the expression of the human growth hormone. Cytosolic and apoplastic-accumulated human growth factor (hGH) has appeared to have the toxic impacts on *N*. *benthamiana* while no negative effect has been observed when the same protein has been targeted to the chloroplast^62,63^.

Transient expression is the known technique for the rapid production (3–5 days) of the recombinant protein in comparison to the stable transformation (6–12 months). Having a higher expression level for a specific pharmaceutical protein is an added value. In our work, the expression level of mtPA has been as high as 10 μgg^−1^ fresh leaf weight or 1.36% of total soluble protein in *N*. *tabacum*. However, Hahn and Masoumi Asl have shown that the accumulation of tPA using the nuclear transformation has been about 0.0014% TSP and 0.026–0.01 μgg^−1^ of the leaf fresh weight, respectively^15,16^. Moreover, Abdoli-Nasab *et al*. (2013) has shown that *N*. *tabacum* carrying the transgenic chloroplast had increased the expression of tPA up to 1% TSP^17^.

In conclusion, our results have shown that despite the presence of the mutation in proximity to the critical site of the catalytic domain, the produced recombinant mtPA fragments have a strong *in vitro* activity in comparison to the commercial Alteplase proteins. However, it is more likely that choosing the appropriate plant species should be considered for the pharmaceutical protein expression^64^. The expression level of mtPA has been ameliorated using P19 virus-encoded gene silencing suppressor in *N*. *tabacum*. On the contrary, the silencing suppressor protein 19 has had an adverse impact on the transiently expressed mtPA in *N*. *benthamiana*. As the transient expression of tPA is a very cost benefit process in terms of the time and expression level, more information on the compatibility among the gene of interest, genetic enhancement tools, and *Nicotiana* species background could be helpful for its application in the pharmaceutical industry.

## Electronic supplementary material

Supplementary figures

Video S1. Surface representation of the interaction between the native form of tPA and plasminogen: the position of Serine and Glycine relative to each other and in the interaction interface with argi

Video S2. Surface representation of the interaction between the mutated form of tPA and plasminogen: the position of Serine and Glutamate relative to each other and in the interaction interface with a
