# Supplementary material for: In silico and in vivo analyses of the mutated human tissue plasminogen activator (mtPA) and the antithetical effects of P19 silencing suppressor on its expression in two Nicotiana species
Source: Sci Rep. 2018 Sep 19;8:14079. doi: 10.1038/s41598-018-32099-6 (PMC6145930; doi:10.1038/s41598-018-32099-6)

In silico and *in vivo* analyses of the mutated human tissue plasminogen activator (mtPA) and the antithetical effects of P19 silencing suppressor on its expression in two *Nicotiana* species

Mahshid Amiri[1], Mokhtar Jalali-Javaran[1]*, Raheem Haddad[2], Parastoo Ehsani[3*]

1 Department of Biotechnology and Plant Breeding, Faculty of Agriculture, Tarbiat Modares University (TMU), Tehran, Iran. m_jalali@modares.ac.ir
2 Agricultural Biotechnology Department, Imam Khomeini International University, Qazvin, Iran

3 Department of Molecular Biology, Pasteur Institute of Iran (IPI), Tehran, Iran. p_ehsani@yahoo.com

Figure S1. Sequence alignment of the cloned sequence and the sequence with accession number I01047. Alignment was generated by MegAlign 5.00 (Lasergene 7, DNASTAR Inc., Madison, WI, USA).

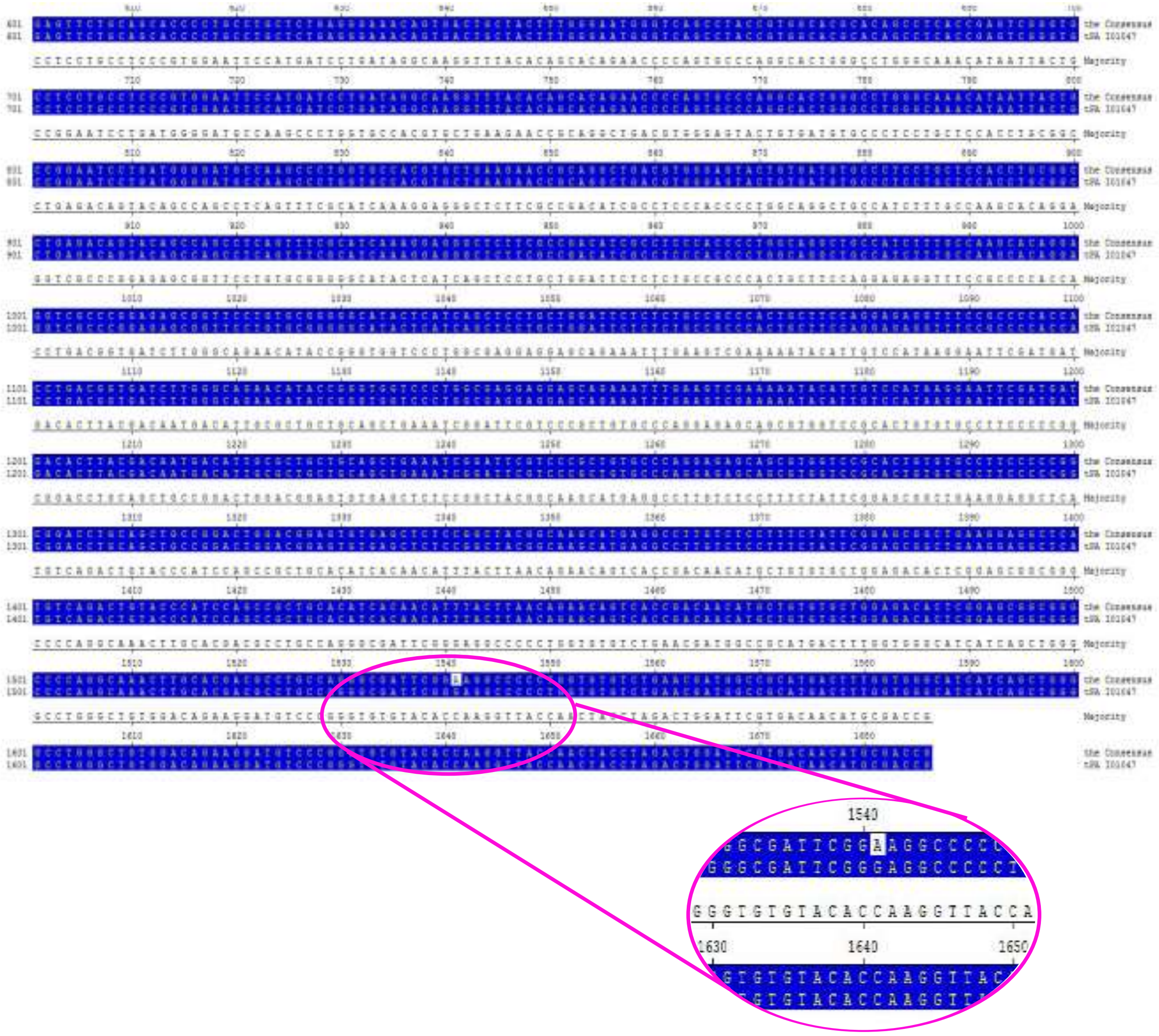

Figure S2. The protein Blast of the translated cloned sequence, the first line (Query) concerns the results of the cloned sequence and the second line pertains to the sequence with accession number AAH95403.1.

Plasminogen activator, tissue [Homo sapiens]

Sequence ID: AAH95403.1 **Length:** 562 **Number of Matches:** 1

Range 1: 1 to 562 GenPept Graphics ▼ Next Match ▲ Previous Match

Score	Expect	Method	Identities	Positives	Gaps
1162 bits(3005)	0.0	Compositional matrix adjust.	558/562(99%)	560/562(99%)	0/562(0%)

```
Query  1    MDAMKRGLCCVLLLCGAVFVSPSQEIHARFRRGARSYQVICRDEKTQMIYQQHQSWLRPV  60
            MDAMKRGLCCVLLLCGAVFVSPSQEIHARFRRGARSYQVICRDEKTQMIYQQHQSWLRPV
Sbjct  1    MDAMKRGLCCVLLLCGAVFVSPSQEIHARFRRGARSYQVICRDEKTQMIYQQHQSWLRPV  60

Query  61   LRSNRVEYCWCNSGRAQCHSVPVKSCSEPRCFNGGTCQQALYFSDFVCQCPEGFAGKCCE  120
            LRSNRVEYCWCNSGRAQCHSVPVKSCSEPRCFNGGTCQQALYFSDFVCQCPEGFAGKCCE
Sbjct  61   LRSNRVEYCWCNSGRAQCHSVPVKSCSEPRCFNGGTCQQALYFSDFVCQCPEGFAGKCCE  120

Query  121  IDTRATCYEDQGISYRGTWSTAESGAECTNWNSSALAQKPYSGRRPDAIRLGLGNHNYCR  180
            IDTRATCYEDQGISYRGTWSTAESGAECTNWNSSALAQKPYSGRRPDAIRLGLGNHNYCR
Sbjct  121  IDTRATCYEDQGISYRGTWSTAESGAECTNWNSSALAQKPYSGRRPDAIRLGLGNHNYCR  180

Query  181  NPDRDSKPWCYVFKAGKYSSEFCSTPACSEGNSDCYFGNGSAYRGTHSLTESGASCLPWN  240
            NPDRDSKPWCYVFKAGKYSSEFCSTPACSEGNSDCYFGNGSAYRGTHSLTESGASCLPWN
Sbjct  181  NPDRDSKPWCYVFKAGKYSSEFCSTPACSEGNSDCYFGNGSAYRGTHSLTESGASCLPWN  240

Query  241  SMILIGKVYTAQNPSAQALGLGKHNYCRNPDGDAKPWCHVLKSRRLTWEYCDVPSCSTCG  300
            SMILIGKVYTAQNPSAQALGLGKHNYCRNPDGDAKPWCHVLKSRRLTWEYCDVPSCSTCG
Sbjct  241  SMILIGKVYTAQNPSAQALGLGKHNYCRNPDGDAKPWCHVLKSRRLTWEYCDVPSCSTCG  300

Query  301  LRQYSQPQFRIKGGLFADIASHPWQAAIFAKHRRSPGERFLCGGILISSCWILSAAHCFQ  360
            LRQYSQPQFRIKGGLFADIASHPWQAAIFAKHRRSPGERFLCGGILISSCWILSAAHCFQ
Sbjct  301  LRQYSQPQFRIKGGLFADIASHPWQAAIFAKHRRSPGERFLCGGILISSCWILSAAHCFQ  360

Query  361  ERFPPHHLTVILGRTYRVVPGEEEQKFEVEKYIVHKEFDDDTYDNDIALLQLKSDSSRCA  420
            ERFPPHHLTVILGRTYRVVPGEEEQKFEVEKYIVHKEFDDDTYDNDIALLQLKSDSSRCA
Sbjct  361  ERFPPHHLTVILGRTYRVVPGEEEQKFEVEKYIVHKEFDDDTYDNDIALLQLKSDSSRCA  420

Query  421  QESSVVRTVCLPPADLQLPDWTECELSGYGKHEALSPFYSERLKEAHVRLYPSSRCTSQH  480
            QESSVVRTVCLPPADLQLPDWTECELSGYGKHEALSPFYSERLKEAHVRLYPSSRCTSQH
Sbjct  421  QESSVVRTVCLPPADLQLPDWTECELSGYGKHEALSPFYSERLKEAHVRLYPSSRCTSQH  480

Query  481  LLNRTVTDNMLCAGDTRSGGPQANLHDACQGDSEGPLVCLNDGRMTLVGIISWGLGCGQK  540
            LLNRTVTDNMLCAGDTRSGGPQANLHDACQGDS GPLVCLNDGRMTLVGIISWGLGCGQK
Sbjct  481  LLNRTVTDNMLCAGDTRSGGPQANLHDACQGDSGGPLVCLNDGRMTLVGIISWGLGCGQK  540

Query  541  DVPGVYTKVTNYLDWIRDNMRP  562
            DVPGVYTKVTNYLDWIRDNMRP
Sbjct  541  DVPGVYTKVTNYLDWIRDNMRP  562
```

Figure S3. ERRAT plot diagram of the selected model.

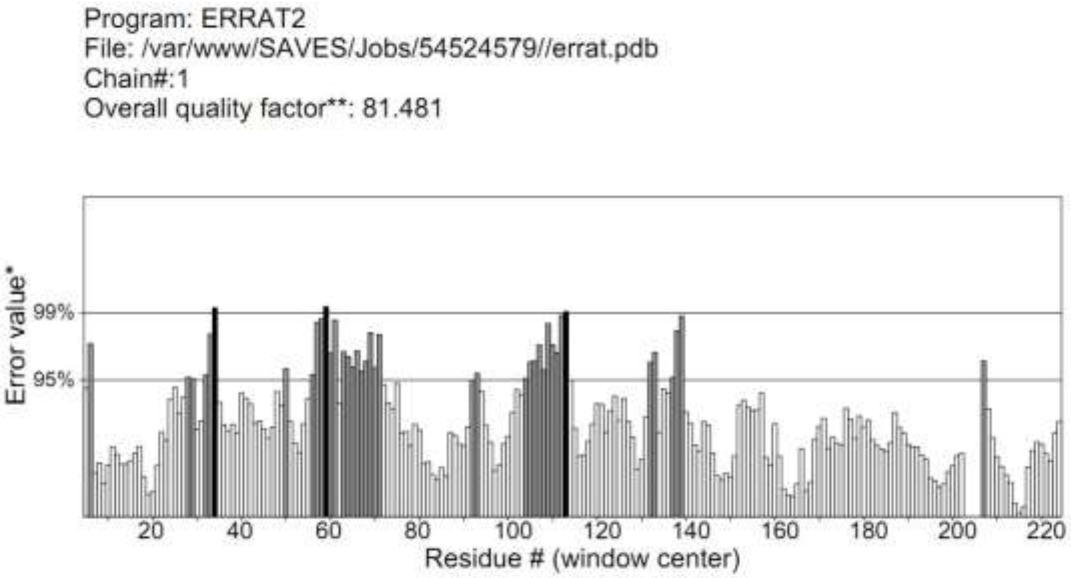

Figure S4. Graph plot of the selected model by Verify3D server.

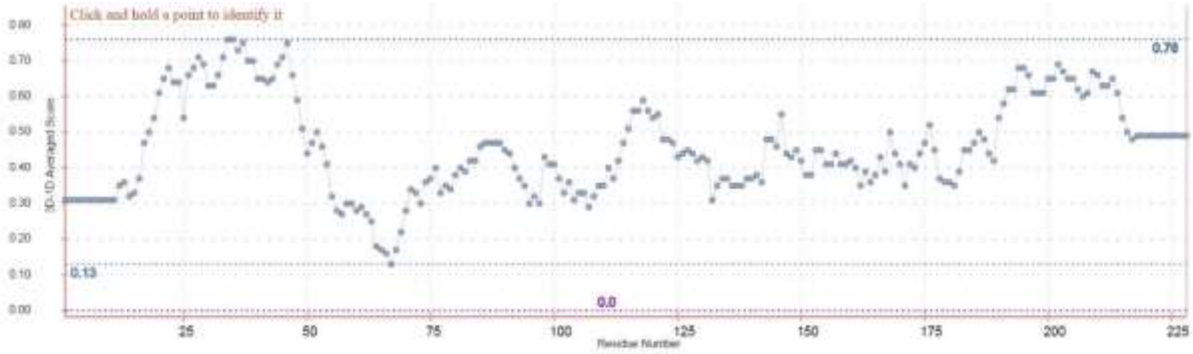

Figure S5.Full length gel of RT-PCR amplification of tissue plasminogen activator on RNA extracted from agroinjected leaves. M: 100 bp plus DNA ladder, C^-: negative control (water template), C^-_R: negative control (extracted RNA), C^+: positive control (pTRAC-ERH-tPA vector), W_T and W_B: agroinjected leaves with *Agrobacterium* containing pTRA-ERH vector in *N. tabacum* and *N. benthamiana*, respectively, 1 and 2: agroinjected leaves with *Agrobacterium* harbouring pMA2 in *N. tabacum* and *N. benthamiana*, respectively. Letters were put in by Microsoft word insertion tools.

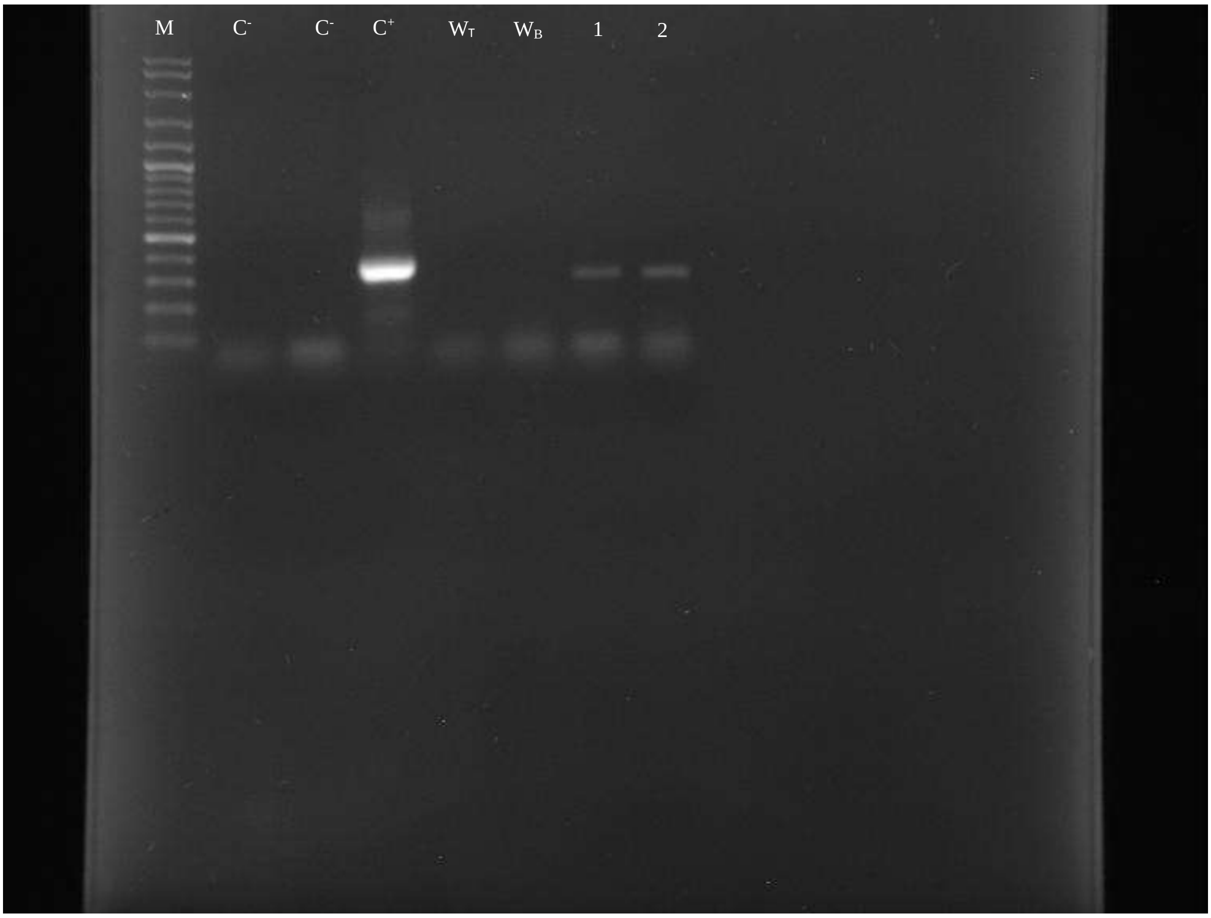

Figure S6. The original figures of dot blot analysis which are cropped in the manuscript to show the differences better; the letters were put in the blot for better undrestanding by Microsoft word tools, the original of every photo (without letter and more copping) was inserted under the respective photo. C^+: positive control (Alteplase protein), W_T and W_B: agroinjected leaves with *Agrobacterium* containing pTRA-ERH vector in *N. tabacum* and *N. benthamiana*, respectivelyt, T2-P19 and B2-P19: tPA+P19 treatment in *N. tabacum* and *N. benthamiana* diluted to OD600=0.2, respectively. T3 and B3: tPA treatment in *N. tabacum* and *N. benthamiana* diluted to OD600=0.3, respectively, and T3-P19 and B3-P19: tPA+P19 treatment in *N. tabacum* and *N. benthamiana* diluted to OD600=0.3, respectively.

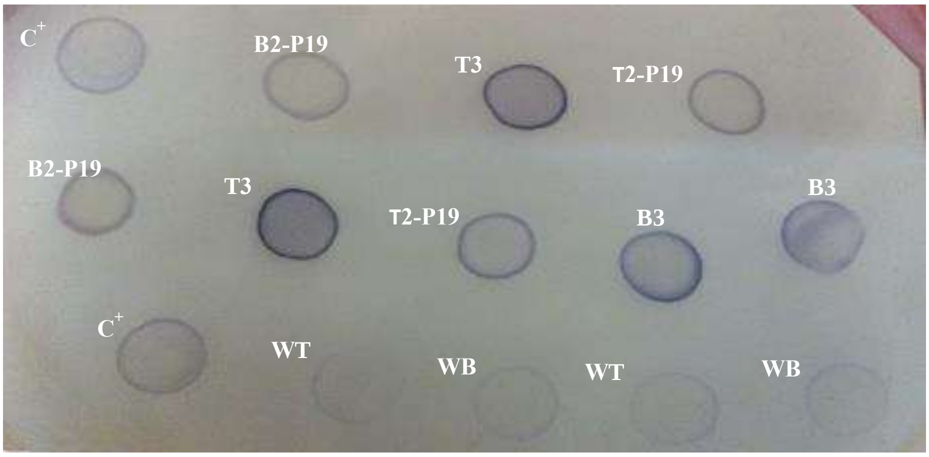

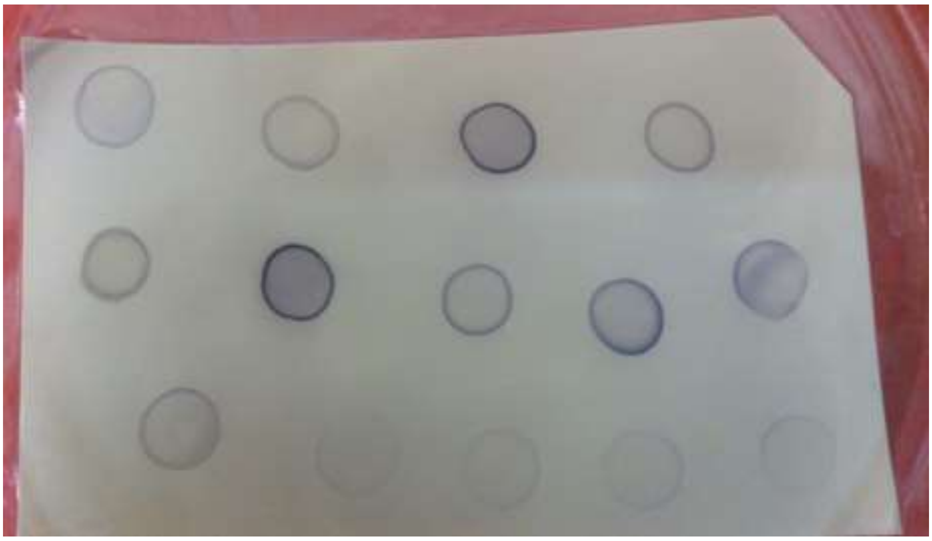

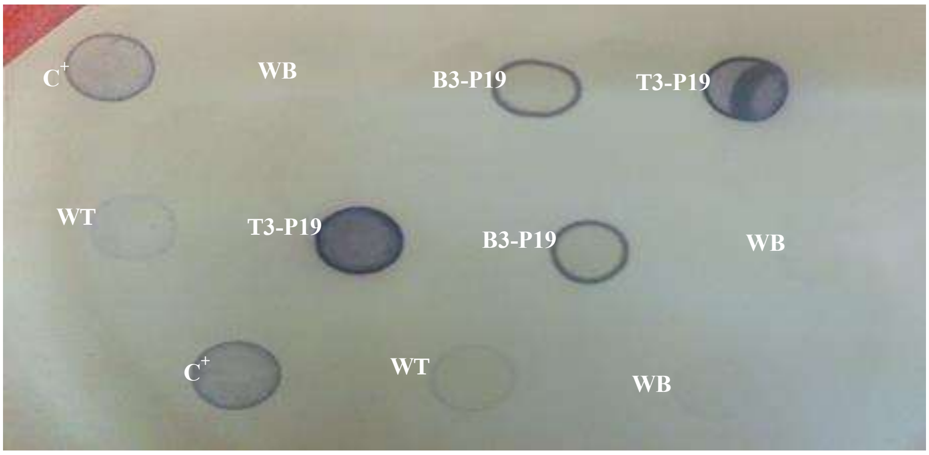

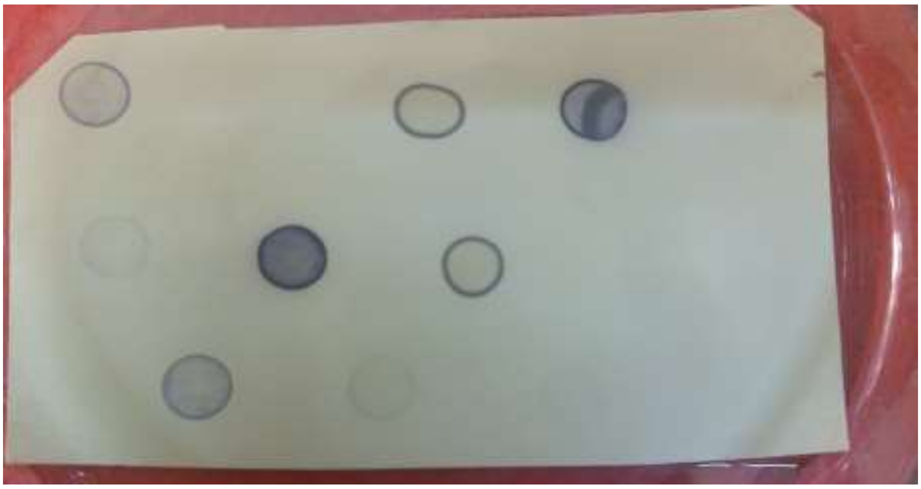

Figure S7. The original figures of western blott analysis with tPA-specific antibody which is cropped in the manuscript to show the differences better. C^+: positive control (Alteplase protein), W_T and W_B: agroinjected leaves with *Agrobacterium* containing pTRA-ERH vector in *N. tabacum* and *N. benthamiana*, respectivelyt, T2-P19 and B2-P19: tPA+P19 treatment in *N. tabacum* and *N. benthamiana* diluted to OD600=0.2, respectively. T3 and B3: tPA treatment in *N. tabacum* and *N. benthamiana* diluted to OD600=0.3, respectively, and T3-P19 and B3-P19: tPA+P19 treatment in *N. tabacum* and *N. benthamiana* diluted to OD600=0.3, respectively. The letters were put on the blot for better undrestanding by Microsoft word tools, the original blot (without letters) was inserted under it.

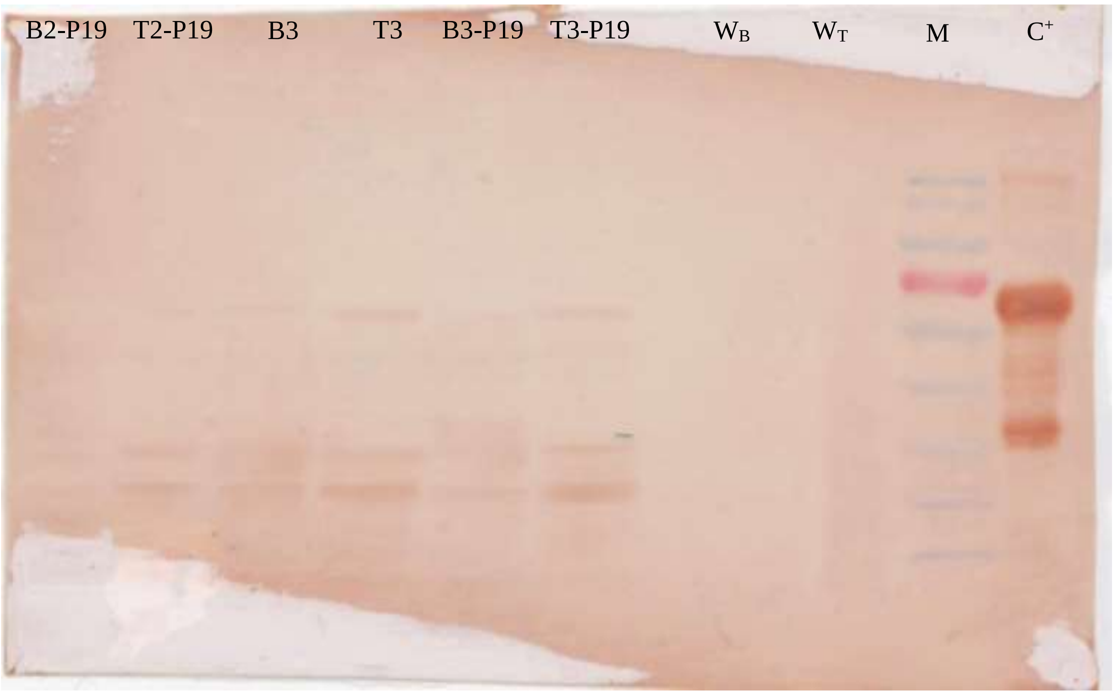

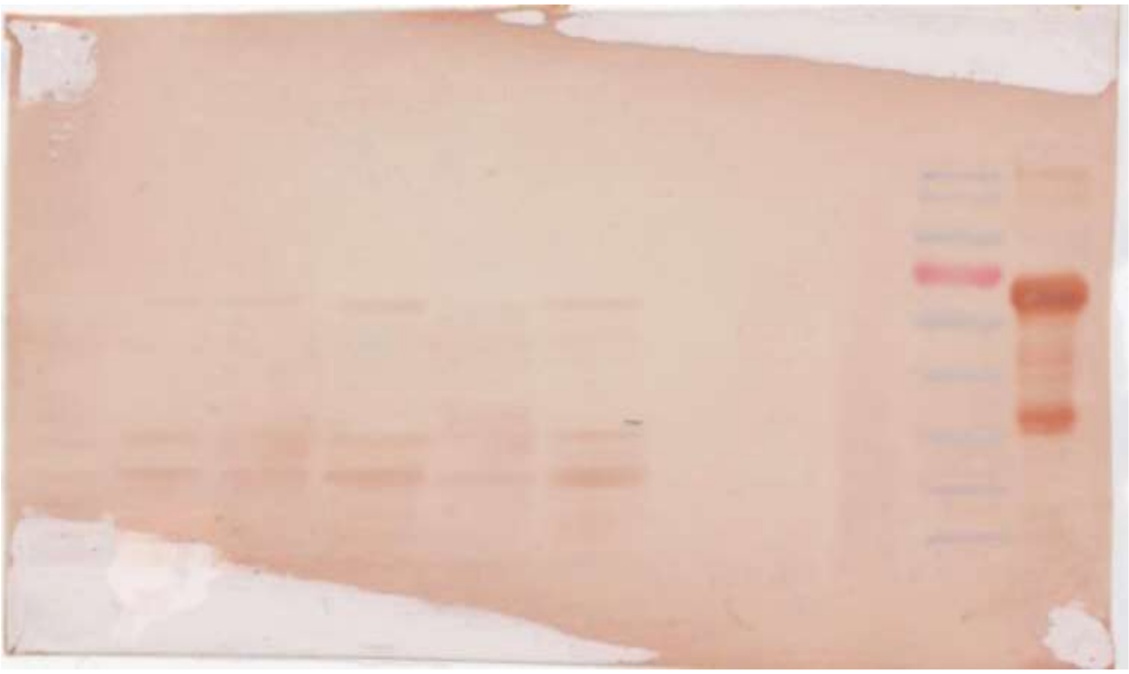

Figure S8. The original figure of Zymography assay which is cropped and its colour is changed in the manuscript to show the differences better. C^+: positive control (Alteplase protein), W_T and W_B: agroinjected leaves with *Agrobacterium* containing pTRA-ERH vector in *N. tabacum* and *N. benthamiana*, respectivelyt, T2-P19 and B2-P19: tPA+P19 treatment in *N. tabacum* and *N. benthamiana* diluted to OD600=0.2, respectively. T3 and B3: tPA treatment in *N. tabacum* and *N. benthamiana* diluted to OD600=0.3, respectively, and T3-P19 and B3-P19: tPA+P19 treatment in *N. tabacum* and *N. benthamiana* diluted to OD600=0.3, respectively. The letters were put on the blot for better undrestanding by Microsoft word tools, the original blot (without letters, color change and cropping) was inserted under it.

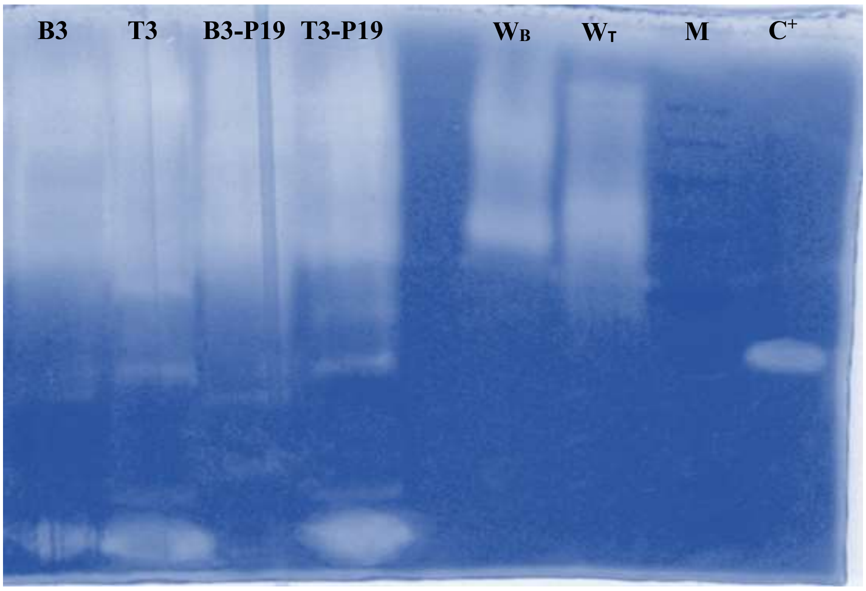

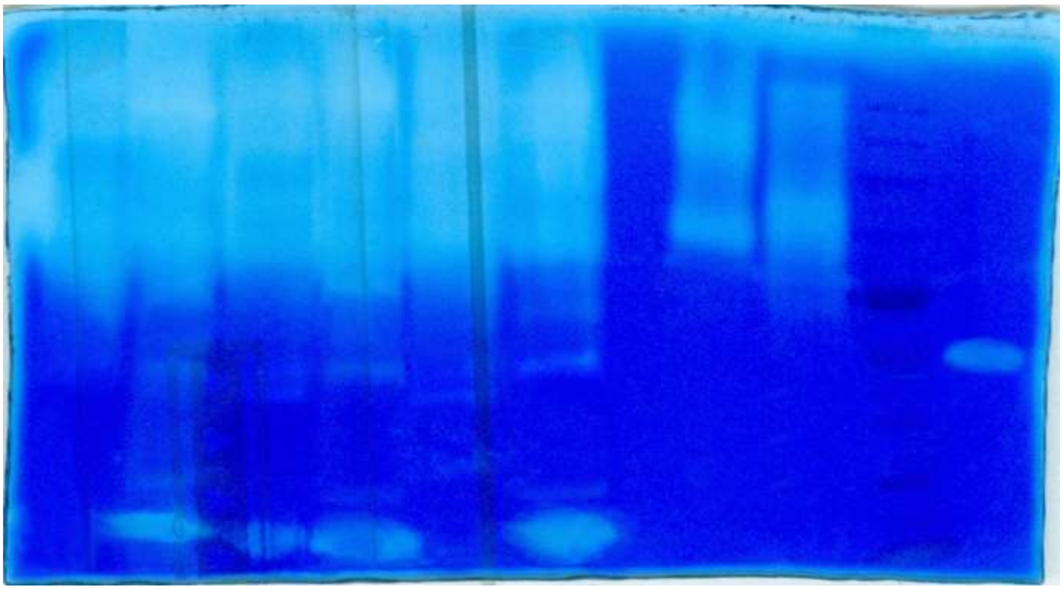

Supplement: Supplementary file 1 — Supplementary figures [file 41598_2018_32099_MOESM1_ESM.pdf]
